# Effects of Race, Cardiac Mass, and Cardiac Load on Myocardial Function Trajectories from Childhood to Young Adulthood: The Augusta Heart Study

**DOI:** 10.1161/JAHA.119.015612

**Published:** 2021-01-18

**Authors:** Gaston Kapuku, Melissa Howie, Santu Ghosh, Vishal Doshi, Michael Bykhovsky, Brittany Ange, James D. Halbert, Vincent Robinson, Zsolt Bagi, Gregory Harshfield, Varghese George

**Affiliations:** ^1^ Departments of Medicine Georgia Prevention Institute Medical College of Georgia Augusta University Augusta GA; ^2^ Department of Pediatrics Medical College of Georgia Augusta University Augusta GA; ^3^ Department of Population Health Sciences Medical College of Georgia Augusta University Augusta GA; ^4^ Department of Physiology Medical College of Georgia Augusta University Augusta GA; ^5^ Department of Leadership and Applied Psychology Adler University Chicago IL

**Keywords:** cardiac function, cardiovascular risk, circumferential end‐systolic stress, growth curve model, left ventricular mass, longitudinal cohort, midwall fractional shortening, Hypertension, Myocardial Biology, Risk Factors

## Abstract

**Background:**

The overall goal of this longitudinal study was to determine if the Black population has decreased myocardial function, which has the potential to lead to the early development of congestive heart failure, compared with the White population.

**Methods and Results:**

A total of 673 subjects were evaluated over a period of 30 years including similar percentages of Black and White participants. Left ventricular systolic function was probed using the midwall fractional shortening (MFS). A longitudinal analysis of the MFS using a mixed effect growth curve model was performed. Black participants had greater body mass index, higher blood pressure readings, and greater left ventricular mass compared with White participants (all *P*<0.01). Black participants had a 0.54% decrease of MFS compared with White participants. As age increased by 1 year, MFS increased by 0.05%. As left ventricular mass increased by 1 g, MFS decreased by 0.01%. As circumferential end systolic stress increased by 1 unit, MFS decreased by 0.04%. The MFS trajectories for race differed from early age to young adulthood.

**Conclusions:**

Changes in myocardial function mirror the race‐dependent variations in blood pressure, afterload, and cardiac mass, suggesting that myocardial function depression occurs early in childhood in populations at high cardiovascular risk such as Black participants.

**Nonstandard Abbreviations and Acronyms**
cESS: circumferential end‐systolic stress
DBP: diastolic blood pressure
HR: heart rate
LVM: left ventricular mass
MFS: midwall fractional shortening
SBP: systolic blood pressure
TPR: total peripheral resistance

**Clinical Perspective**

**What Is New?**

- This multiethnic and longitudinal study assesses the development of cardiovascular risk factors from children with family histories of cardiovascular diseases (ie, essential hypertension and/or premature myocardial infarction).
- Left ventricular contractility was evaluated from early childhood through young adulthood using the midwall fractional shortening.
- The midwall fractional shortening trajectories for race differed from early age to young adulthood, and longitudinal trajectory of midwall fractional shortening changes with age might prove to be a useful tool for assessment of early interventions in a multiethnic cohort.

**What Are the Clinical Implications?**

- Our results indicate that changes in myocardial function mirror the race‐dependent variations in blood pressure, afterload, and cardiac mass.
- Our findings suggest that myocardial function depression occurs early in childhood in populations at high cardiovascular risk such as Black participants.
- This study calls for earlier intervention to curb the epidemic of heart failure in populations at high cardiovascular risk such as Black participants.

The Augusta Heart study and other population studies^1^, ^2^, ^3^ have demonstrated the role of cardiac mass as a preclinical marker of cardiovascular disease. Increased cardiac mass leads to congestive heart failure, which remains one of the leading causes of cardiovascular morbidity and mortality.^4^ However, the relationship between cardiac mass and heart function is not completely elucidated.

Traditional estimation of systolic function uses ultrasound technique, nuclear imaging, and cardiac magnetic resonance imaging to determine the ejection fraction. Altogether, these methods may overestimate systolic function in individuals with remodeling of the left ventricle and/or concentric hypertrophy due to normal aging, blood pressure elevation, and/or obesity. Ejection fraction fails to capture the progressive decline of cardiac function during the life span, raising the possibility of its inadequacy to effectively capture the myocardial function over time.^5^ Taking into account the established prognostic value of left ventricular (LV) systolic dysfunction, an appropriate assessment of cardiac systolic function using both chamber and myocardial function is warranted because even a subtle reduction of LV systolic function predicts increased likelihood of developing congestive heart failure.^6^

Global heart function is carried out mainly by circumferentially aligned muscle fibers located in the middle layer of the LV wall.^7^, ^8^, ^9^ Less attention has been paid to these fibers, which contribute to shortening of the LV short axis and ejection. In this study, we measured the midwall fractional shortening (MFS) as a way to probe the contraction of these fibers. MFS may identify subclinical decline of systolic performance even in the presence of a normal chamber function, as measured by ejection fraction. Myocardial structure and function are regulated by load conditions. Increased afterload induces myocardial hypertrophy and decreased pump function. Subtle decreases in MFS are associated with increased likelihood of developing congestive heart failure.^10^

Numerous data have demonstrated sex and ethnic differences in cardiac mass and geometry, but thorough evaluations of the differences in cardiac pump function are scarce.^11^, ^12^, ^13^, ^14^ The CARDIA (Coronary Artery Risk Development in Young Adults) study^11^ reported demographic changes of cardiac mass only during a 5‐year follow‐up, which may or may not reveal significant changes in systolic function. In our prospective cohort, which spanned from childhood to young adulthood, we sought to longitudinally characterize myocardial contractility and evaluate the contribution of age, race, sex, height, blood pressure, heart rate, total peripheral resistance (TPR), and circumferential end systolic stress (cESS) to the chronological changes of myocardial function. We hypothesized that cardiac function would be decreased in individuals with greater cardiac mass, such as Black participants, and that this alteration would be detected by myocardial function markers rather than by ejection fraction alone.

## Methods

### Subjects

Subjects in this study were participants in the Augusta Heart Study, which is a longitudinal study of the development of cardiovascular risk factors in children with verified family histories of cardiovascular diseases (ie, essential hypertension and/or premature myocardial infarction).^1^, ^15^, ^16^, ^17^, ^18^

This study included 673 subjects measured multiple times with a combined 4596 observations. Of these 673 subjects, 334 (49.6%) were male participants, 339 (50.4%) were female participants, 352 (52.3%) were of White ancestry, and 321 (47.7%) were of Black ancestry.

### Procedures

The study was approved by Augusta University’s Institutional Review Board committee. The protocol has been previously published.^17^ The data that support the findings of this study are available from the corresponding author upon reasonable request. Informed consent was obtained during the first visit. Because of the longitudinal design that the subjects agreed to, it was not necessary to obtain new consent for each visit. All anthropometric and hemodynamic evaluations were conducted by a female research assistant of the same race as the subject. Subject’s height (in centimeters) and weight (in kilograms) were measured without shoes with a Health‐O‐Meter medical scale, which was calibrated daily. The subject was escorted to a quiet temperature‐controlled room (20°C to 22°C) and fitted with equipment for recording blood pressure and heart rate (Dinamap model 1846 SX, Critikon Inc) and cardiac output using thoracic bioimpedance (NCCOM‐3, Bo Med Medical Manufacturing Ltd), as previously described.^19^

### Hemodynamic Evaluations

Stroke volume and cardiac output were measured every successive 12 QRS intervals while the Dinamap device was inflating and calculating pressure. TPR was calculated with the use of concurrently derived systolic blood pressure (SBP) and diastolic blood pressure (DBP) and cardiac output as follows: [(SBP + DBP)/3]/CO, expressed in Wood units (mm Hg/L per minute).

### Echocardiographic Studies

To assess LV contractile function, cESS, MFS, and MFS ratio were calculated according to established formulas. Specifically, we calculated cESS at the midwall level of the left ventricle as an index of afterload using a cylindrical model, as follows ^14^:

$$\text{cESS}=\frac{\text{SBP}\times {\left(\text{LVIDS}/2\right)}^{2}\times \left\{1+\frac{{\left(\text{LVIDS}/2+\text{LVPWS}\right)}^{2}}{{\left(\text{LVIDS}/2+\text{LVPWS}/2\right)}^{2}}\right\}}{{\left(\text{LVIDS}/2+\text{LVPWS}\right)}^{2}‐{\left(\text{LVIDS}/2\right)}^{2}},$$

where LVIDS is LV internal diameter in systole and LVPWS is LV posterior wall thickness in systole.

MFS was calculated following the method described by de Simone et al,^14^ as, MFS=(LVIDD + LVPWD/2 + IVSD/2)−(LVIDS + Hs/2)/(LVIDD + LVPWD/2 + IVSD/2), where LVIDD is the LV internal diameter in diastole, LVPWD is the LV posterior wall thickness in diastole, and Hs is the LV inner shell myocardial thickness at the end of systole, taking into account the epicardial migration of midwall during systole in a spherical model. To evaluate midwall LV performance independently of afterload, MFS ratio was calculated as the ratio between MFS calculated from the echocardiographic measurement and the value predicted for a given level of cESS.^19^ For the prediction of MFS, we incorporated a comprehensive model that also included race, sex, age, height, body mass index (BMI), TPR, SBP, DBP, heart rate, and LV mass (LVM), in addition to cESS. We used the concomitant R to R interval captured on echocardiocardiogram instead of the Dinamap‐derived heart rate in the model, because it measures the heart rate more accurately.

Reliability quality control checks were performed on a random sample of 20% of the subjects. Intrarater and interrater coefficients of variation for all cardiac structures assessed were less than 10%, comparable to previous studies in our laboratory^16^, ^20^, ^21^, ^22^ and to other published findings.^23^, ^24^

### Statistical Analysis

A longitudinal analysis of the MFS using a mixed effect growth curve model was performed. The fixed effect of a factor in the model represents the mean of the trajectory pooling of all the individuals within the sample, and the random effect represents the variance of the individual subject’s trajectories around the group means. To account for the variability of MFS among visits, we used a random intercept model, with race, sex, age, height, BMI, TPR, SBP, DBP, R to R interval, LVM, and cESS as fixed effects.

Variable selection is one of the most important steps in the process of statistical model building for the understanding of the underlying process that generates the data and for improving the performance of the predictors. Traditional variable selection approaches for longitudinal data are based on some information criteria, such as the Akaike information criterion or the Bayesian information criteria. However, these involve an exhaustive search over all submodels, and these methods are not always practically feasible in terms of the computational cost and time when the number of submodels is large. In our case, the total number of submodels is 2048, which is large and hence to avoid the computational burden, we considered a more sophisticated variable selection method, known as least absolute shrinkage and selection operator.^25^

Least absolute shrinkage and selection operator uses cross‐validation technique to determine the optimal model that has a minimum prediction error. In our case, a 5‐fold cross‐validation was used to find the best model in terms of prediction error, and the resulting final model included race, age, height, SBP, DBP, TPR, R to R interval, LVM, and cESS (see Appendix).

## Results

### Initial Visit Findings

Table 1 lists anthropometric, hemodynamic, and echocardiographic data at the initial visit, by race. Based on these data, Black participants had higher weight, BMI, SBP, DBP, TPR, and LVM and lower MFS compared with White participants.

**Table 1:** Subjects' Characteristics at Initial Visit by Race

	Black	White	*P* Value
Demographics			
Number of Subjects	321	352	0.2321
Age, y	15.4 ± 3.0	15.1 ± 3.33	0.2083
Anthropometric Measures			
Height, cm	164.6 ± 9.8	163.0 ± 12.7	0.0526
Weight, kg	68.6 ± 22.5	61.2 ± 20.4	<0.0001
Body mass index, kg/m^2^	25.0 ± 7.2	22.6 ± 5.8	<0.0001
Hemodynamic Measures			
Systolic blood pressure, mm Hg	111.3 ± 10.7	107.8 ± 8.8	<0.0001
Diastolic blood pressure, mm Hg	59.5 ± 7.1	57.2 ± 5.9	<0.0001
Total peripheral resistance, mm Hg (min/L)	16.2 ± 3.9	14.7 ± 3.8	<0.0001
Heart rate, bmp	69.1 ± 12.0	70.2 ± 15.2	0.2942
Echocardiographic Measures			
Left ventricular mass, g	127.6 ± 35.8	118.8 ± 37.7	0.0013
Ejection fraction, %	62.3 ± 7.2	62.3 ± 6.9	0.9912
MFS, %	18.6 ± 2.4	19.0 ± 2.5	0.0348
MFS ratio	97.4 ± 11.6	98.2 ± 12.6	0.3595
Circumferential end‐systolic stress, 10^3^dyne/cm^2^	136.5 ± 29.5	138.5 ± 30.9	0.5493

MFS indicates midwall fractional shortening.

Male participants had higher height, SBP, TPR, cESS, LVM, and MFS ratio and lower BMI, DBP, and heart rate compared with female participants (Table 2).

**Table 2:** Subjects' Characteristics at Initial Visit by Sex

	Male	Female	*P* Value
Demographics			
Number of Subjects	334	339	0.8472
Age, y	15.3±3.1	15.2±3.2	0.5509
Anthropometric Measures			
Height, cm	167.0±13.0	160.5±8.6	<0.0001
Weight, kg	65.7±21.7	63.8±21.8	0.1588
Body mass index, kg/m^2^	23.1±5.6	24.4±7.4	0.0164
Hemodynamic Measures			
Systolic blood pressure, mm Hg	111.7±10.3	107.3±8.9	<0.0001
Diastolic blood pressure, mm Hg	57.6±6.3	59.0±6.7	0.0120
Total peripheral resistance, mm Hg (min/L)	15.8±4.1	15.1±3.6	0.0067
Heart rate, bmp	67.0±12.4	72.3±14.6	<0.0001
Echocardiographic Measures			
Left ventricular mass, g	133.4±39.5	112.9±31.3	<0.0001
Ejection fraction, %	62.2±6.7	65.5±7.3	0.5594
MFS, %	19.0±2.4	18.7±2.5	0.1580
MFS ratio	99.3±12.0	96.4±12.1	0.0022
Circumferential end‐systolic stress, 10^3^dyne/cm^2^	141.8±30.1	133.3±29.7	0.0002

MFS indicates midwall fractional shortening.

### Relationship Among Cardiac Load, Structure, and MFS Trajectories

**Figure 1:**
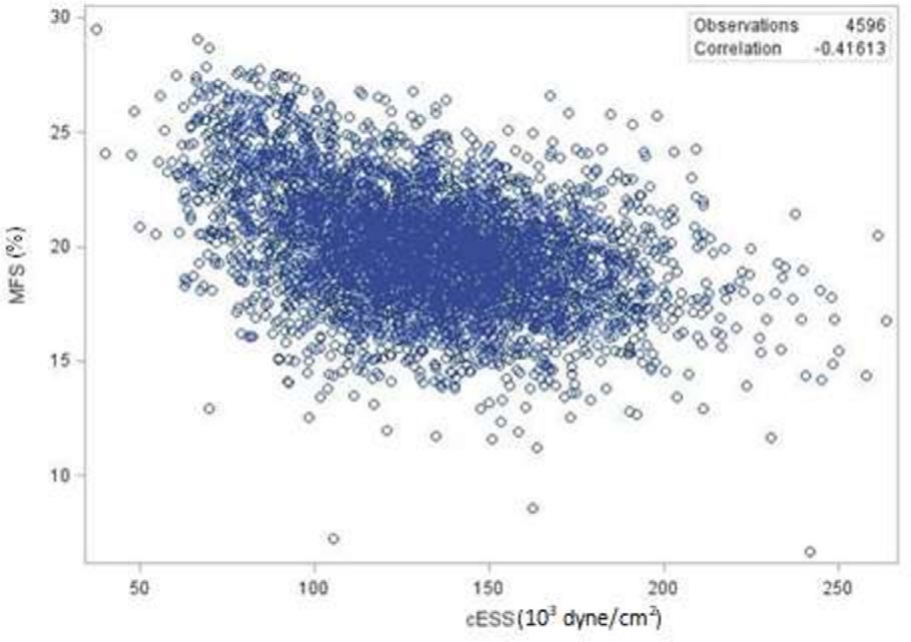
Relationship between cESS and MFS. cESS indicates circumferential end‐systolic stress; and MFS, midwall fractional shortening.

**Figure 2:**
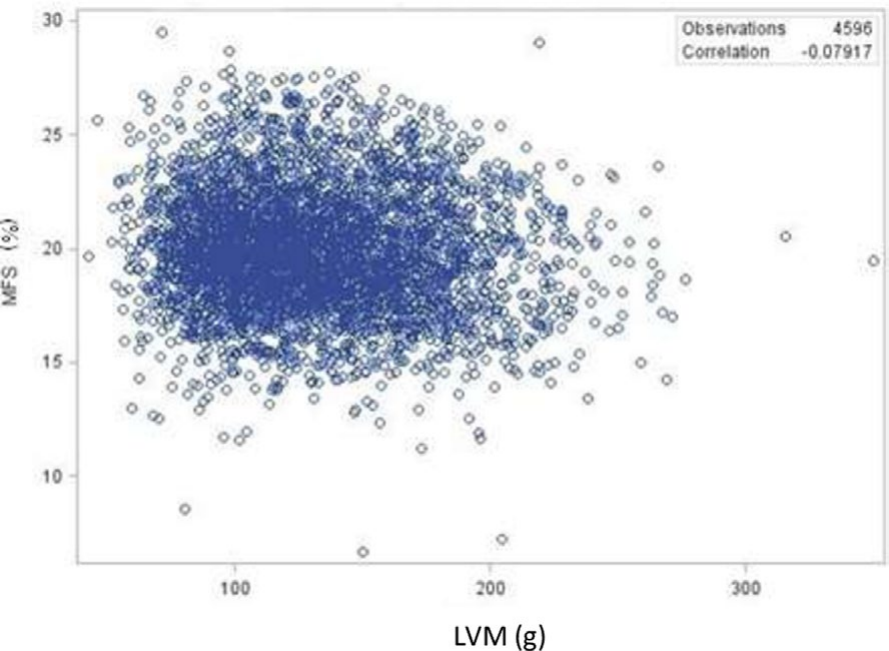
Relationship between LVM and MFS. LVM indicates left ventricular mass; and MFS, midwall fractional shortening.

**Figure 3:**
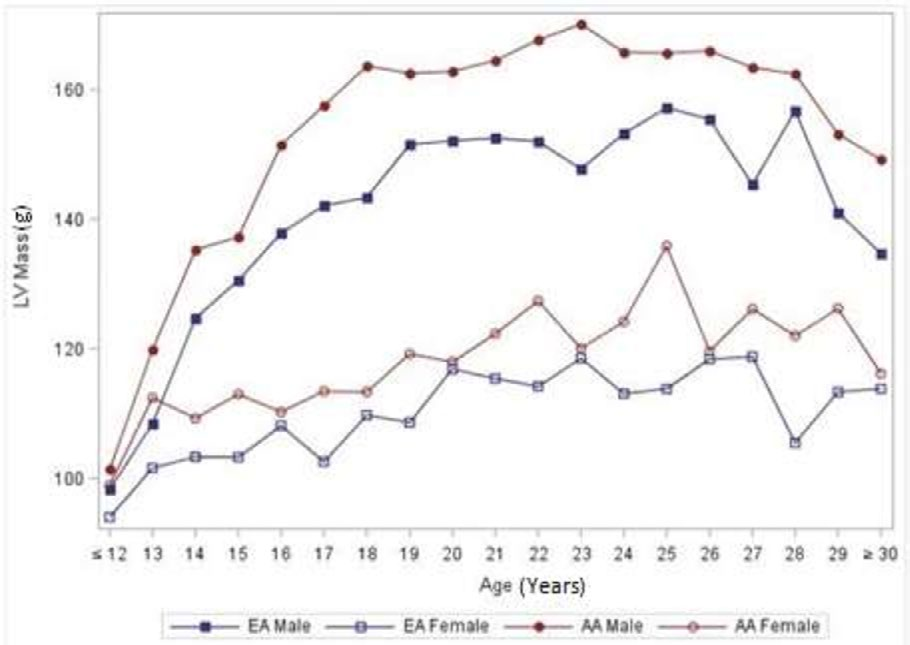
Mean LVM. EA, European American (White); LV, left ventricular; and LVM, left ventricular mass.

Figure 1 depicts the relationships between MFS and cESS. There was a moderate association between cESS and MFS. Higher cESS was associated with lower MFS. There was no association between LVM and MFS (Figure 2). The mean values of raw LVM did not differ in early childhood by ethnicity. The ethnicity and sex differences became apparent in midadolescence and remained fairly stable with age (Figure 3). LVM was lower in female participants than in male participants (*P* < 0.0001). It was also lower in White participants than in Black participants (*P* < 0.0001).

**Figure 4:**
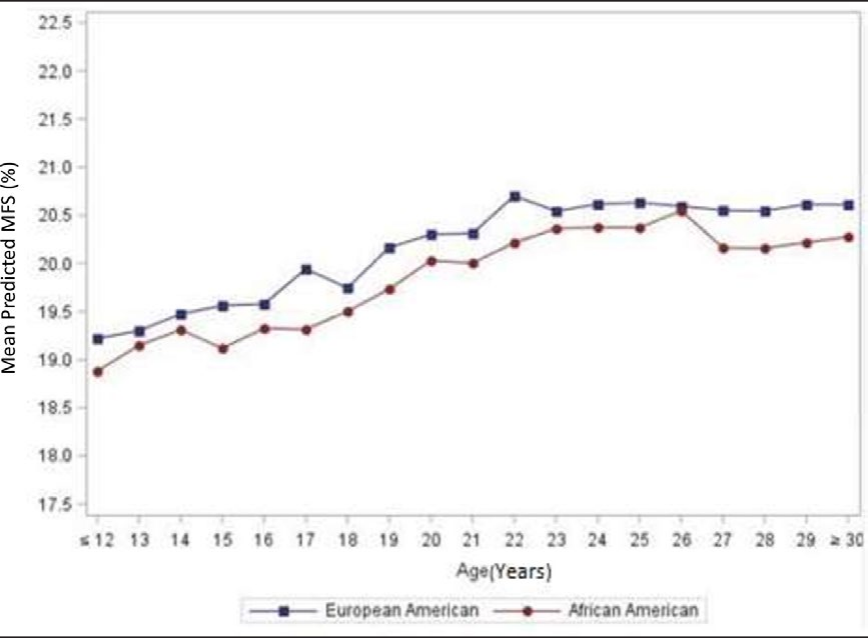
Trajectory of predicted MFS by race. MFS indicates midwall fractional shortening.

Age appears to have both linear and quadratic effects on MFS. Figure 4 shows the predicted values of MFS from childhood to young adulthood. MFS generally increases with age for White and Black participants. The predicted MFS trajectories differed from early age to young adulthood. Black participants had lower predicted MFS compared with White participants.

### Estimated Prediction Model for MFS Trajectory

The parameter estimates of the fixed effects of the prediction model for MFS using the least absolute shrinkage and selection operator technique are given in Table 3. These estimates for the predictors are the estimated change in MFS corresponding to a unit change in that predictor, when all the other predictors are held constant. Precisely, Black participants had a 0.54% decrease of MFS compared with White participants. As age increased by 1 year, MFS increased by 0.05%. As LVM increased by 1 g, MFS decreased by 0.02%. Other MFS enhancing predictors included height, SBP, DBP, and heart rate reflected by R to R interval.

**Table 3:** Parameter Estimates in the Midwall Fractional Shortening Prediction Model

Effect	Estimate	Standard Error	*P* Value
Intercept	14.04	0.794	<0.0001
Race	‐0.54	0.094	<0.0001
Age	0.05	0.008	<0.0001
Height	0.04	0.005	<0.0001
Systolic blood pressure	0.04	0.005	<0.0001
Diastolic blood pressure	0.03	0.007	0.0001
Total peripheral resistance	‐0.11	0.009	<0.0001
R to R interval	2.16	0.250	<0.0001
Left ventricular mass	‐0.02	0.001	<0.0001
Circumferential end‐systolic stress	‐0.04	0.001	<0.0001

On the other hand, as cESS increased by 1 unit, MFS decreased by 0.04%. Other MFS depressing predictors included TPR and LVM. As expected, Black and female participants had lower predicted MFS compared with White and male participants, respectively, over the observation period (Table 4).

**Table 4:** Midwall Fractional Shortening Least Squared Means in Model Based on Age, Race, and Sex Only

Sex	Race	Estimate	Standard Error	*P* Value
Female		20.13	0.068	<0.0001
Male		19.63	0.069	
	Black	19.56	0.069	<0.0001
	White	20.20	0.069	

## Discussion

The aim of this study was to evaluate race differences in the cardiac function from early childhood through young adulthood. To the best of our knowledge, this prospective study is the first to examine the life span changes of myocardial function in a healthy cohort. We report changes in the MFS over time, with significant differences between Black and White participants. Our findings validate and expand longitudinally the cross‐sectional findings of de Simone et al, demonstrating an age‐dependent change in MFS as well as an inverse relationship between MFS and cESS.^14^, ^26^ The higher the afterload, the lower the myocardial systolic function.

Physiologic and pathologic remodeling of the heart occurs with normal aging process owing to normal growth and changes in loading conditions.^27^ It is accepted that an increase in cardiac size is associated with normal adaptative systolic function or inadequate adaptation expressed as decreased contractile function. Usually, identifying these subtle and progressive changes requires sensitive probing tools. However, volumetric measures such as ejection fraction or fractional shortening are typically used, which may fail to identify preclinical systolic dysfunction.^28^, ^29^ Hence, circumferential myocardial functional assessment has been proposed to better characterize the contribution of myocardial mechanics to the pump function.^30^ Herein, we found no association between cardiac mass and MFS whereas there was a moderate association between afterload measure and MFS, suggesting that cardiac function deterioration depends more on load conditions than the change in wall thickness and cardiac volumes. These anatomical changes reflect a myriad of ultrastructural changes such as myocyte slippage, myocyte hypertrophy, and fibrosis.^27^

Evaluation of our model demonstrates that the MFS increases with age from childhood to adulthood. Predicted LV function as reflected by MFS was lower in Black participants than White participants. These findings mitigate the role of increasing cardiac mass on LV function from childhood to young adulthood. Because our cohort was made with healthy individuals, it could be possible that cardiac function adaptative mechanisms came into play to compensate for increasing afterload experienced during young adulthood. Further evaluation might enable us to recognize the transition from such adaptive to maladaptive remodeling and progression to systolic dysfunction.

Increased blood pressure burden in Black participants may be an important cause of ethnicity differences in cardiac mass. We previously reported that Black participants experience more stress‐induced sodium retention than White participants.^31^ Long‐lasting stress and high salt intake occur more frequently in Black participants, together with gene‐environment predisposition to higher sodium consumption and retention. This may explain ethnicity discrepancies in ambulatory blood pressure.^32^ Ethnic differences in ambulatory blood pressure patterns translate in different patterns of cardiac structure. We demonstrated that daytime, nighttime, and 24‐hour ambulatory SBP are positively related to indexed LVM 2.3 years later. Olutade et al^33^ observed nondipping in Black participants and linked it with a 6‐fold higher prevalence of concentric remodeling, suggesting that nighttime blood pressure is a strong determinant of cardiac remodeling.

Genetic factors rather than afterload or cardiac mass may also contribute to myocardial function depression. In pediatric populations X chromosome linked mutations (eg, G4.5 gene) are related to the development of isolated ventricular noncompaction, which is an arrest of the intrauterine squeezing of the myocardial fibers and meshwork.^34^, ^35^ This LV feature is associated with depressed cardiac function. In adults, the isolated ventricular noncompaction genetic profile is associated with systolic dysfunction and poor clinical outcome.^36^, ^37^ It has been reported that Black athletes have higher trabeculation in LV compared with White athletes.^38^ LV trabeculation may be misdiagnosed as LV noncompaction. This LV pattern represents an adaptative response to a chronic increase in preload and afterload associated with exercise. In our longitudinal LV measures of healthy youths and young adults no more than 3 prominent trabeculations in the left ventricle were observed. Also, all individuals with suspected cardiomyopathies were excluded.

Overall, the trajectories of cESS mirrored those of MFS, evoking a possible stronger link than that of MFS to cardiac mass. The greater correlation for the first linkage than the second could evoke such possibility even though it does not imply causation.

The important strength of this study is the usage of a multiethnic cohort, representative of the population of the southeastern United States that is known to have high percentage of inhabitants with high risk of developing cardiovascular diseases^39^, ^40^ However, our findings need to be interpreted within the scope of some limitations. First, the assessment of myocardial function was based only on ultrasound‐based determination of ejection fraction and midwall fractional shortening. Adding assessment of LV strain and measure of diastolic function derived from tissue Doppler would permit a better characterization of myocardial function.^40^ Second, the longitudinal basis of this study allowed for potential long‐term attrition from the study. To offset the loss of these subjects, new participants of equivalent age, race, and sex were included in the study following individual subject departure. This reinclusion of subjects within the study allowed for continued investigation of a normal, representative population. Finally, no probing biomarkers precluded the inference of potential underlying mechanisms involved in the differential myocardial function.

## Conclusions

Our findings support an important role of cardiac load on myocardial function trajectories from childhood to early adulthood. Black participants showed a decreased myocardial performance when compared with White participants. With afterload being the central driver of myocardial performance during normal aging, we thus identify one possible mechanism that may explain how cardiac performance may deteriorate in time in populations with high cardiovascular risk.

Our reported changes in myocardial function among adolescents and young adults mirror the well‐known ethnicity‐ and sex‐dependent variations in blood pressure, afterload, and cardiac mass. As these changes are demonstrated to occur in youth, our findings call for earlier intervention to curb the epidemic of heart failure in populations at high cardiovascular risk, such as Black participants.

## Appendix

Random intercept and mixed effects variables were race, age, htcm, supsbp_, supdbp_, suptpr_, r_r, lvmass, and cess2. The residual maximum likelihood estimation method was used for the covariance parameters.^41^ Usually, the least absolute shrinkage and selection operator has several limitations. It may select the wrong variables when there are strong correlations and the resulting estimates are biased toward zero. To address these issues we refitted the selected model and report the estimates in Table 3. Finally, we computed Spearman correlation matrix among the predictors. The correlations between the predictors were weak and in a few cases, the correlations between the predictors were moderate. Thus, the least absolute shrinkage and selection operator producer should not be affected because of the correlations between predictors.

## Sources of Funding

None.

## Disclosures

None.
